# Atypical skeletal manifestations of rickets in a familial hypocalciuric hypercalcemia patient

**DOI:** 10.1038/boneres.2017.1

**Published:** 2017-06-27

**Authors:** Bo Wu, Ou Wang, Yan Jiang, Mei Li, Xiaoping Xing, Weibo Xia

**Affiliations:** 1 Department of Endocrinology, Key Laboratory of Endocrinology, Ministry of Health, Peking Union Medical College Hospital, Peking Union Medical College, Chinese Academy of Medical Sciences, Beijing, China

## Abstract

Familial hypocalciuric hypercalcemia (FHH) is caused by inactivating mutations in the calcium-sensing receptor (*CaSR*) gene. The loss of function of *CaSR* presents with rickets as the predominant skeletal abnormality in mice, but is rarely reported in humans. Here we report a case of a 16-year-old boy with FHH who presented with skeletal manifestations of rickets. To identify the possible pathogenic mutation, the patient was evaluated clinically, biochemically, and radiographically. The patient and his family members were screened for genetic mutations. Physical examination revealed a pigeon breast deformity and X-ray examinations showed epiphyseal broadening, both of which indicate rickets. Biochemical tests also showed increased parathyroid hormone (PTH), 1,25-dihydroxyvitamin D, and elevated ionized calcium. Based on these results, a diagnosis of FHH was suspected. Sequence analysis of the patient’s *CaSR* gene revealed a new missense mutation (c.2279T>A) in exon 7, leading to the damaging amino change (p.I760N) in the mature CaSR protein, confirming the diagnosis of FHH. Moreover, the skeletal abnormities may be related to but not limited to vitamin D abnormity. Elevated PTH levels and a rapid skeletal growth period in adolescence may have also contributed. Our study revealed that rickets-like features have a tendency to present atypically in FHH patients who have a mild vitamin D deficiency, and that *CaSR* mutations may have a partial role in the pathogenesis of skeletal deformities.

## Introduction

Familial hypocalciuric hypercalcemia (FHH) is an inherited disorder of mineral homeostasis that is transmitted as an autosomal dominant trait. FHH is characterized biochemically by lifelong modest elevation of the serum calcium concentration with a relatively mismatched hypocalciuria and normal or mildly elevated circulating parathyroid hormone (PTH) level that is not suppressed by the hypercalcemia.^1,2^ Individuals with FHH are usually asymptomatic, and the disorder is considered benign. FHH is associated with heterogeneous inactivating mutations in the calcium-sensing receptor (*CaSR*) gene.^3^ The CaSR protein is a G-protein-coupled receptor that belongs to family (or class) C, which includes seven exons and is mainly expressed in parathyroid and renal tubule cells, as well as in bones.^4^

*CaSR* helps maintain a nearly constant levels of extracellular calcium (Ca_o_^2+^) in the blood and other extracellular fluids. By sensing even minute changes in the Ca_o_^2+^ from its normal level, *CaSR* modulates the functions of key tissues, especially the parathyroid glands and the kidneys.^5^ In the parathyroid glands, *CaSR* senses perturbations in the Ca_o_^2+^ level of only a few percent and then responds to the hypocalcemia by increasing PTH secretion, stabilizing mRNA and inducing the proliferation of parathyroid cells. In the kidneys, *CaSR* stimulates Ca^2+^ reabsorption in the cortical thick ascending limb in response to hypocalcemia, and 1-hydroxylation of 25-hydroxyvitamin D_3_ [25(OH)D_3_] in the proximal renal tubules.^6,7^

*CaSR* is also expressed in the skeleton, but the importance of *CaSR* in bone formation and resorption remains somewhat controversial. Studies in mice with a conditional knockout of the *CaSR* gene have indicated that *CaSR* has key roles in osteoblasts.^1^ The skeleton could mediate PTH independently with the homeostatical uptake or release of calcium during induced hypocalciuria, actions that may involve the *CaSR* gene. The calcium-sensing receptor is present in the skeleton, and the absence of exon 5 results in defective mineralization of cartilage and bone, which produces rickets as the predominant skeletal abnormality in mouse models.^8^ In case reports, human FHH caused by inactive mutations of *CaSR* rarely manifested as rickets. A recent report described a patient who developed severe adolescent rickets due to vitamin D deficiency.^2^ Another report considered that vitamin D deficiency modulates the severity of FHH.^3^

In this study, we report an FHH patient with typical skeletal manifestations. The patient demonstrated a fracture with transient hypophosphatemia, which confounded the plausible initial diagnosis of rickets based on the clinical findings. In addition to hypocalciuria, increased PTH and the an ultimately slightly elevated serum calcium eventually led to consideration of FHH. The identification of a *CaSR* mutation confirmed the FHH diagnosis. The patient also presented with a mild vitamin D deficiency, but that alone could not completely explain the manifestation of rickets. We proposed the possibility that a patient with *CaSR* mutation may tend to present with skeletal phenotype of rickets under conditions of vitamin D deficiency. Further, elevated PTH and pubescent skeletal growth and development modulate the manifestation of rickets in FHH patients.

## Materials and methods

### Subject

A 16-year-old boy who was admitted to our hospital for knee pain that had lasted 2 years and who had suffered a femur fracture 4 months prior with subsequent nonunion presented with signs of rickets. The patient underwent clinically, biochemically, and radiologically evaluations. He was the child of a non-consanguineous couple, without any craniofacial or skeletal abnormality recorded in his family history.

### Biochemical parameters

Fasting blood samples were collected, and serum was stored at −80 °C for measurement. The concentrations of serum calcium (Ca), serum phosphate (Pi), serum creatinine (Scr), serum alkaline phosphatase (ALP), 24-h urinary phosphate and urinary calcium were measured using the routine methods available at the central laboratory of Peking Union Medical College Hospital, an in-hospital laboratory accredited for quality supervision standards.

25-hydroxyvitamin D [25(OH)D], serum intact PTH, and β C-terminal telopeptide of bone type I collagen concentrations were measured with an automated electrochemiluminescence system (Roche Diagnostics, Basel, Switzerland). Serum 1,25-dihydroxyvitamin D_3_ [1,25(OH)_2_D_3_] was measured with a 1,25-dihydroxyvitamin D [1,25(OH)_2_D] ^125^I RIA kit (DiaSorin, Stillwater, USA). Intact fibroblast growth factor 23 (FGF23; Kainos Laboratories Inc., Tokyo, Japan), sclerostin (Biomedica Medizinprodukte GmbH, Vienna, Austria), receptor activator for nuclear factor-κ B ligand (Biomedica Medizinprodukte GMBH), and osteoprotegerin (Biomedica Medizinprodukte GMBH) were measured using a two-site enzyme-linked immunosorbent assay according to the manufacturer’s instructions.

### Molecular genetic analysis

#### Targeted next-generation sequencing

Genomic DNA of the patient and his parents was extracted from peripheral leukocytes using a QIAamp DNA Blood Mini kit (QIAamp DNA; Qiagen, Hilden, Germany). A customized targeted enrichment kit (Roche NimbleGen, Madison, USA) was used to capture the splice sites of all exons and the immediate intron-flanking sequences of 722 genes associated with rare genetic skeletal disorders or that were involved in the pathways related to skeletal development. A previously described pipeline was used for targeted sequence capture and for sequencing library preparation.^9–11^ Sequencing (PE101) was performed using an Illumina HiSeq2000 platform (Illumina, San Diego, USA) according to the standard manual.

#### Read mapping and variant detection, annotation, and interpretation

Illumina Pipeline (version 1.3.4) was used for image analysis, error estimation, base calling, and generation of the primary sequence data. The primary sequence data were analyzed using GAEA software, which was developed in-house at the Beijing Genomics Institute (BGI) and integrated the modules for quality control of raw data, reads alignment, SNV calling and INDEL calling. The annotation of SNVs and INDELs was accomplished using in-house scripts. The clinical significance of variants was interpreted according to the guidelines from American College of Medical Genetics and Genomics.^12^

#### Sequence analysis of the human *CaSR* gene

Sanger sequencing was used to confirm the potential mutations identified by targeted next-generation sequencing. PCR was performed to cover all exons and 50 bp on both sides of the exon–intron boundaries of *CaSR*. The samples were sequenced with an automated sequencer, and sequence alignment was performed using the basic local alignment search tool (BLAST) from the National Center for Biotechnology Information database. The bioinformatics tool PolyPhen was used to predict the protein function.

### Radiography

Radiographic studies were performed in the Department of Radiology of Peking Union Medical College Hospital. Plain X-rays of both hands and lateral films of the thoracolumbar vertebra and femur were taken.

### Ethics statement

Approval for this study was obtained from the local ethics committee in the Department of Scientific Research at Peking Union Medical College Hospital. The patient and his family provided informed consent to participate in this study.

## Results

### Clinical features

A 16-year-old Chinese boy was admitted to our hospital for a 2-year duration of knee pain. The patient also presented with a femur fracture that had occurred 4 months prior with subsequent nonunion, which rendered him wheelchair-bound.

The patient was born via natural labor, had been breast-fed, and had never developed rickets-like symptoms when he was a child. He started to sit and walk at the appropriate age and presented equal or higher height compared with his peers. He had no difficulty in feeding, walking, or running. His growth and pubertal development were normal. There was no history of delayed tooth eruption, bowed legs, waddling, tetany, or skeletal deformities either. The remaining developmental milestones were within the normal limits.

From the age of 10 years, he practiced the sport of fencing and gradually developed gonalgia at approximately 12–13 years old, which was considered a consequence of fencing. Then, he began to complain of more severe and localized pain in the knee related to exercise, which was relieved by rest. He also felt the occasional backache, which was accompanied by minor weakness.

The patient gradually developed the clinical signs of rickets (pectus carinatum) until 14 years of age. He presented with a slight, ameliorative pain after minor trauma to the right lower extremity when bicycling, without impaired physical mobility. However, two days later he felt severe localized pain around the right knee with aggravated symptoms and arthrocele. X-ray examination was performed at a local hospital and revealed fractures of the right distal femur and proximal fibula. An external fixator with traction did not resolve the problem, and the patient presented with delayed union and angulation deformities of the long bones. Laboratory evaluation showed hypophosphatemia and normal serum calcium, increased alkaline phosphatase, procollagen I N-terminal peptide, and C-terminal telopeptides of bone type I collagen.

He was treated for 2 months with oral 1,25(OH)_2_D 0.25 μg per day, and calcium carbonate 1 200 mg per day as the basic treatment for osteoporosis. Then, the patient underwent surgery for the placements of an internal fixator that was performed on the right femur. Interestingly, the patient’s biochemical indicators showed increased and nearly normalized serum phosphate one to two months later.

After being admitted to our hospital, a laboratory examination of the patient showed a normal serum phosphate concentration. The parents stated that the patient’s family history was negative for hypophosphatemia, rickets, osteomalacia, and other metabolic bone diseases. His height and weight were within the calculated mid-range (height of 168 cm, between the median and 1 s.d. of the same age and gender population; weight of 50 kg, between −1 s.d. and the median), and his body mass index was 17.7 kg·m^−2^.

Physical examination revealed a pigeon breast deformity, possible rachitic rosary, and bilateral widening of the wrists and ankles. Dental examination showed no dental caries or periodontal abscess, and the patient did not present with bowing of the lower extremities or other skeletal deformity.

X-ray examinations revealed signs of rickets, such as epiphyseal broadening in the distal femur, the proximal fibula, and the proximal phalanx, as well as a lower bone density.

Laboratory evaluation revealed a normal to lower serum ionized calcium of 1.03–1.23 mmol·L^−1^ (normal range, 1.13–1.23 mmol·L^−1^) with a serum level of total calcium of 2.36–2.46 mmol·L^−1^ (normal, 2.13–2.70 mmol·L^−1^), whereas the serum concentration of phosphorus was normal or mildly increased (1.48–1.77 mmol·L^−1^; normal range, 0.81–1.45 mmol·L^−1^). The patient showed a mildly high level of alkaline phosphatase of 287 –394 U·L^−1^ (normal range, 42–390 U·L^−1^), which led to the suspected diagnosis of rickets. The serum concentration of 25(OH)D_3_ was 17.6 ng·mL^−1^ (normal, 8–50 ng·mL^−1^), which seemed low but was considered normal in the Chinese population. The intact PTH concentrations (90.7–117 pg·mL^−1^; normal, 12–65 pg·mL^−1^) were increased, and the patient also showed an extraordinarily elevated serum concentrations of 1,25(OH)_2_D: 213.95 pg·mL^−1^ (normal range, 19.6–54.3 pg·mL^−1^). Therefore, the vitamin D deficiency and likely secondary hyperparathyroidism were never effectively treated during the patient’s course of treatment (to avoid vitamin D overdosage). Moreover, the 24-h urine calcium of the patient was significantly decreased, ranging from 0.31 to 0.82 mmol (normal, 2.5–7.5 mmol per 24 h), which was not consistent with rickets. Bone turnover markers were also assessed. The receptor activator for nuclear factor-κ B ligand concentration was significantly elevated, up to 0.688 pmol·L^−1^, and serum osteoprotegerin concentrations were decreased to 1.3 pmol·L^−1^. β C-terminal telopeptide of type I collagen was also increased, which indicated increased osteoclast activity. However, FGF23 and sclerostin concentrations were normal, reaching 4.096 1 pg·L^−1^ and 26.5 pmol·L^−1^, respectively.

One year later after hospitalization, the patient was pain-free with no aggravated skeletal deformity. Biochemical examination showed a mildly elevated serum ionized calcium of 1.32 mmol·L^−1^ (normal range, 1.13–1.23 mmol·L^−1^), with a total calcium concentration of 2.48 mmol·L^−1^. The 24-h urine calcium (0.4 mmol) was significantly lower compared with the normal range.

In all, during the patient’s course of the disease, he presented with laboratory values of an elevated serum ionized calcium, a continuously high PTH levels and a significantly decreased 24-h urine calcium, and the clinical diagnosis of FHH was highly suspected and later established. Negative radiological result of the parathyroid and significantly repetitive low 24-h urine calcium suggested that the diagnosis was unlikely to be primary hyperparathyroidism.

### Imaging features

**Figure 1:**
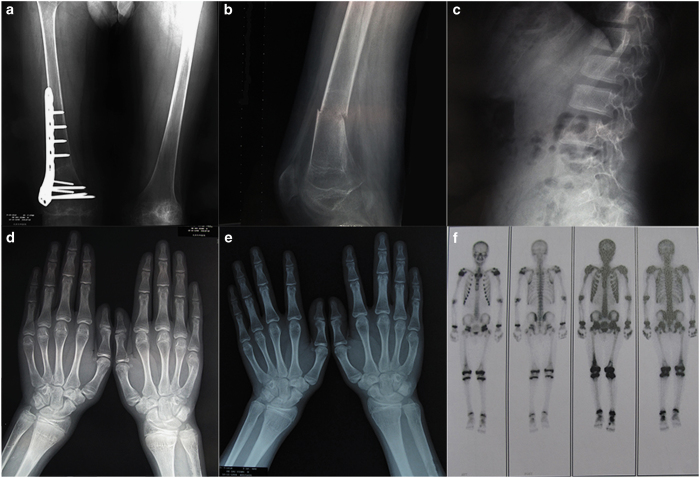
Radiographs and bone scans of a Chinese patient with familial hypocalciuric hypercalcemia. Radiographs of (**a**) orthophoria of the femur (postoperation), (**b**) lateral film of the femur (nonunion of fracture), and (**c**) lateral film of the lumbar vertebra showing osteoporosis. X-ray of the hands indicating epiphyseal broadening before (**d**) and after (**e**) epiphyseal closure. (**f**) Bone scan showing no specific foci of abnormal tracer deposition.

X-ray of the hands indicated epiphyseal broadening. X-ray films of the femur and the lumbar vertebra also demonstrated decreased bone density and sparse trabecula of the bones (Figure 1).

Medical imaging results showed that the bone mineral density (BMD) of the lumbar vertebra 1–4 was 0.820 g·m^−2^ (*Z* score, −1.3) and the BMD of the femoral neck was 0.460 g·m^−2^ (*Z* score, −2.7), whereas that of the total femur was 0.467 g·m^−2^ (*Z* score, −3.0). The anteroposterior delayed phase of the bone scan showed no specific foci of abnormal tracer deposition except on the right distal femur (Figure 1f).

One year later, the BMD of the spine at L1–4 had increased to 1.015 g·m^−2^ (*Z* score, 0.6), the BMD of the femoral neck was 0.599 g·m^−2^ (*Z* score, −2.2), and the BMD of the total femoral was 0.401 g·m^−2^ (*Z* score, 3.3). X-ray of the hands still indicated epiphyseal broadening even after the epiphyseal closure.

### Mutation analysis

**Figure 2:**
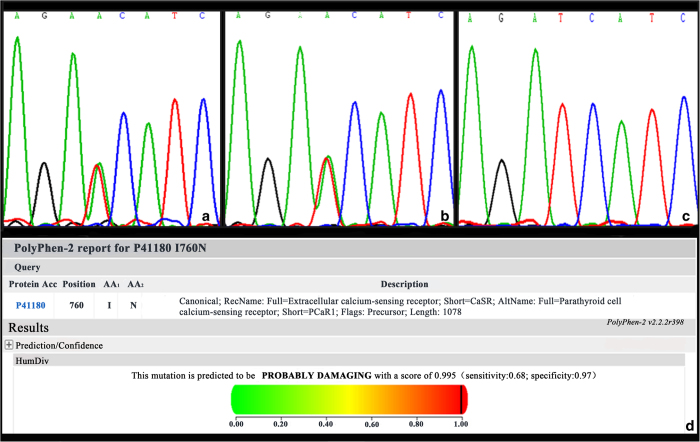
Genetic analysis of *CaSR* in the proband and his parents. (**a**) Mutation analysis revealed a missense mutation of exon 7 (c.2279T>A, p.I760N) on the *CaSR* in the proband and his father (**b**, **c**). Sequences of his mother were normal. (**d**) Prediction of the impact of p.I760N mutation on the structure and function of the CaSR protein using PolyPhen2.

The targeted next-generation sequencing and direct sequencing of the coding regions of genetic hypophosphatemic rickets-related genes were normal. Both the next-generation sequencing and the Sanger sequencing data of amplified genomic DNA showed a heterozygous mutation on exon 7 of *CaSR* (c.2279T>A, p.I760N; NP_000379.2). The patient’s father was found to be a carrier but did not exhibit clinical symptoms of FHH. The sequences of the patient’s mother were normal (Figure 2a–c). We have excluded the single-nucleotide polymorphism reported in The National Center for Biotechnology Information (http://www.ncbi.nlm.nih.gov/). We also performed direct sequencing of *CaSR* in 50 Chinese healthy controls, and 2279 T>A was not found in this group.

### Pathogenicity prediction

The bioinformatics tool PolyPhen (http://genetics.bwh.harvard.edu/pph) was used to predict the effects of the missense mutation on protein function. The HumVar score was 0.913, indicating a possibly damaging mutation (Figure 2d).

## Discussion

We have described a case of FHH with a new heterozygous *CaSR* mutation, I760N, in a patient who did not exhibit any obvious clinical symptom until the age of 16 years when he developed a spontaneous fracture and adolescent rickets. Laboratory evaluation revealed an elevated serum ionized calcium level, a continuously high PTH level, and a significantly decreased 24-h urine calcium, which are consistent with FHH.

**Figure 3:**
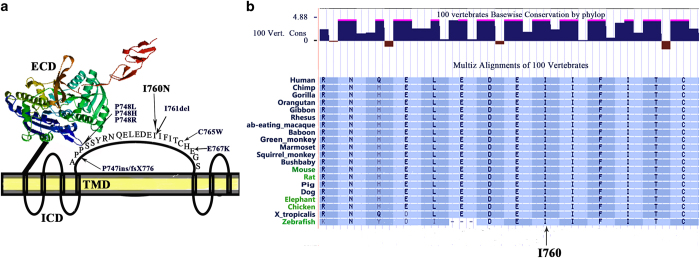
Sites of known mutations and protein sequence alignment in the second extracellular loop of the model of CaSR. (**a**) Extracellular domain (ECD) of CaSR was shown in color diagram according to protein structure homology-modeling server (http://swissmodel.expasy.org/). The seven membrane spanning helices of CaSR are shown, based on hydropathy plot analysis (www.casrdb.mcgill.ca/). (**b**) Protein sequence alignment of the I760N mutation among different species, as given in the NCBI database.

We found a new mutation in exon 7 of *CaSR* in this patient, which is located in an important region of the transmembrane domain (TMD) of CaSR, which contains seven trans-intracellular helices, three relatively short extracellular loops and three intervening intracellular loops. The mutation in this patient was located in the second loop of the TMD. This domain might have a role in the activation of cellular signaling through multiple downstream signaling pathways: Gq/11, Gi/o, and G12/13. These signal pathways are closely related to the pathogenesis of FHH,^13^ because the mutation of GNA11 causes FHH type 2.^14^ In this patient, mutation I760N was located in the second extracellular loop of the TMD, which contains multiple reported mutations (Figure 3). In particular, the reported mutation of I761del is actually equivalent to the mutation of I760del, because the amino acids located in 760 and 761 were both isoleucine. In other words, although I760N was a new mutation, I760del was a known mutation. Moreover, isoleucine was located in the main evagination of the domain and possessed a high conservatism during species evolution (Figure 3b). In light of this information, the isoleucine located in 760 was crucially important for the normal function of CaSR, although function experiments in this location was not performed.

In addition, Chang and Shoback *et al* reported a conditional deletion of exon 7 in the *CaSR* in osteoblasts, leading to severely under-mineralized matrix and osteoid accumulation. A conditional deletion of exon 7 in chrondrocytes produced a rickets-like phenotype with expansion and reduced mineralization of the hypertrophic zone and decreased abundance of mature markers. This result indicated that the mutation in exon 7 of *CaSR* may be functionally significant in the skeleton.^15^ Moreover, the bioinformatics tool PolyPhen indicated a possibly damaging mutation. In addition, I760N was not found in the *CaSR* genes in 100 healthy chromosomal controls, and was not reported as a single-nucleotide polymorphism.

Gene analysis has shown that the mutation location was related to the clinical manifestation. It has been suggested that mutation receptors of this type could more effectively downstream effects in the kidney than in the parathyroid glands, which produces a greater “resistance” to Ca^2+^_o_ in the latter than in the former.^16^ However, little data have focused on the difference between skeletal and other manifestations, and one study reported that there was no relationship between plasma alkaline phosphatase, PTH, and regional BMD in FHH patients, and U-NTx/creatinine ratio was inversely correlated to the BMD of the forearm.^13^ This finding might indicate that the severity in different organs varies.

The role of *CaSR* in bone development *in vivo* has been controversial, although several studies have concluded that it mediates Ca^2+^_o_ sensing in osteoblasts. In 2001, an unexpected skeletal phenotype of rickets was presented in *CaSR*-absent mice, indicating that calcium-sensing receptor was present in the skeleton and its absence resulted in the defective mineralization of cartilage and bone.^1^ However, the lack of apparent skeletal defects in global *CaSR*^−/−^ and *PTH*^−/−^ mice indicated a bone disorder resulted from the direct effects of PTH.^17–19^ Recent studies have focused on the function of *CaSR* in both the chondrocytes in growth plates and osteoblast-lineage cells. For growth plates, *CaSR* has been detected in maturing chondrocytes,^19^ and knockdown of *CaSR* impaired cell differentiation and matrix mineralization.^2,20^ For the osteoblast lineage, transgenic mice confirmed the biological significance of *CaSR* in bone development. After deleting the *CaSR* in early OBs, mice showed multiple skeletal fractures, soft bones, and large amounts of immature osteoid.^2,19^ In adulthood, their bones were severely osteoporotic,^2,21,22^ together with the increased expression of receptor activator of nuclear factor kappa-B ligand and decreased expression of osteoprotegerin. The symptom and bone turnover manifestation were consistent with our patient.^23,24^

However, few reports have discussed the skeletal performance of patients with a *CaSR* mutation, especially in patients with FHH. One case analyzed an older asymptomatic FHH patient who suffered from osteoporosis and fracture with subsequent nonunion,^21^ and whose presentation was consistent with the patient in this study. Other reports have described adolescent rickets of FHH patients, which is mainly attributed to elevated PTH or vitamin D deficiency.^2,3^ However, in this study the vitamin D concentration (13.7–18.6 ng·mL^−1^) was approximated to the mean 25(OH)D level in the Chinese population (19.4±6.4 ng·mL^−1^ and 13.2±5.4 ng·mL^−1^).^25,26^ The elevated PTH, attributed to both FHH and secondary hyperparathyroidism induced by low concentration of 25(OH)D, may contribute to the clinical manifestation of rickets.

In addition, other factors affect the development of rickets. Rickets is not limited to infancy or early childhood; but is also reported in adolescents. However, rickets occur during the period of rapid bone growth, with increased metabolic demands, it may develop long before any physical findings or radiological evidence are obtained.^20,27,28^ Hypocalcemic tetany and limb pain were the most common presenting symptoms in adolescents, whereas radiological evidence was not present in every case.^29,30^ The reported cases of FHH with clinical manifestations of rickets also occurred in adolescence, a period of rapid skeletal growth and mineralization.^2,3^ For this patient, the skeletal pain gradually appeared during his puberty, and his symptoms alleviated after the epiphyseal closure (Figure 1e). Furthermore, PTH directly affects bone mineralization, decreases mineralization in mature cells, and promotes bone turnover, which may lead to osteomalacia.^13,31,32^ There have been cases reported that primary hyperparathyroidism could masquerade as rickets, especially in childhood or in adolescence.^33–35^ The possible explanation is that atypical clinical manifestations of FHH may coexist with vitamin D deficiency, and might be further aggravated by elevated PTH levels. Moreover, there may be deleterious effect of continuously high level of PTH that acts directly on chondrocytes.

## Conclusion

We have reported the case of a 16-year-old male with a heterozygous *CaSR* mutation, who developed skeletal abnormalities of spontaneous fracture with subsequent nonunion, and adolescent rickets. However, the skeletal abnormalities could not be simply explained by the known causes of a vitamin D deficiency or an increased PTH concentration. It has been suggested that rickets-like features may tend to present as uncommon characteristics in FHH patients with mild vitamin D deficiency, pubescent skeletal growth and epiphyseal closure, which may work to modulate the manifestation of rickets. There are some limitations in our study. We did not conduct a functional analysis of the mutant CaSR protein of the patient and could not provide a direct mechanism-based explanation of the relationship between *CaSR* and rickets. However, rickets and fractures have been rarely described in humans, although they have always been present in *CaSR*-absent mice. This case and other previous reports confirmed the clinical performance of rickets and fracture in FHH patients, and we hope to arouse concern about the symptoms of rickets or fractures in adolescent FHH patients with vitamin D deficiency, which might be ignored in clinical practice. Moreover, further studies are needed to determine the accurate function of *CaSR* in maintaining phosphate homeostasis and bone mineralization.
